# Study on the Thermal and Rheological Properties of Nano-TiO_2_-Modified Double Phase Change Asphalt

**DOI:** 10.3390/ma18204799

**Published:** 2025-10-21

**Authors:** Xingming Liu, Xiaojun Cheng, Shanshan Wang, Sishuang Wei, Meng Guo, Shanglin Song, Fukui Zhang

**Affiliations:** 1 Gansu Gannan New Century Road and Bridge Co., Ltd., Gannan 747000, China; 18189693310@163.com; 2 Scientific Observation and Research Base of Transport Industry of Long Term Performance of Highway Infrastructure in Northwest Cold and Arid Regions, Dunhuang 736200, China; 18193106299@163.com; 3 The Key Laboratory of Urban Security and Disaster Engineering of Ministry of Education, Beijing University of Technology, Beijing 100124, China; chengxj@emails.bjut.edu.cn (X.C.); w15872611417@163.com (S.W.); 4 State Key Laboratory of Bridge Engineering Safety and Resilience, Beijing University of Technology, Beijing 100124, China; 5 Expressway Operation Center, Shandong Hi-Speed Group Co., Ltd., Jinan 250101, China; 18615677067@163.com; 6 Gansu Provincial Highway Development Group Co., Ltd., Lanzhou 730070, China; 13993109118@163.com

**Keywords:** phase change materials, temperature regulation effect, rheological properties

## Abstract

In this paper, paraffin-44H (PW-44H) and paraffin-5 (PW-5) were respectively selected as the high/low-temperature phase change core material, and expanded vermiculite (EVM) was selected as the phase change carrier matrix. A high-temperature composite phase change material (CPCM), 44H/EVM, and a low-temperature CPCM, 5/EVM, were prepared by combining melt blending with vacuum adsorption. Nano-TiO_2_ was incorporated as a thermal conductor into the CPCMs to enhance the heat transfer efficiency between the CPCM and asphalt. The heat storage performance, chemical stability, microstructure, and thermal stability of the two CPCMs were studied. The results show that when the dosage of nano-TiO_2_ is 2%, the critical temperature range and heat storage performance of the CPCMs reach the optimum. Among them, the enthalpy value of the phase transition of the high-temperature PCM 44H-nTiO_2_/EVM is 150.8 J/g, and the phase transition occurs over a temperature range of 37.3 °C to 45.9 °C. The enthalpy value of the phase transition of the low-temperature PCM 5-nTiO_2_/EVM is 106.6 J/g, and the phase transition range is −7.9–0.4 °C. Moreover, the incorporation of nano-TiO_2_ increased the thermal conductivity of the high- and low-temperature CPCMs by 47.2% and 51.6%, respectively. Finally, the high- and low-temperature CPCMs were compounded in a 1:1 ratio and mixed into asphalt to obtain a composite double PCM asphalt. The heat storage performance of the original sample asphalt and the double phase change asphalt was investigated by DSC, DSR, and an environmental chamber. The results show that when the dosage of PCM is 20%, compared with the original asphalt, the high-temperature extreme value and the low-temperature extreme value of the double phase change asphalt are reduced by 3.4 °C and 2.1 °C, respectively. The heating rate and cooling rate decreased by 8.5% and 5.6%, respectively, and the rheological properties can meet the requirements of the specifications. It can be seen that the addition of double PCMs can effectively slow down the heating/cooling rate of asphalt, thereby improving the temperature sensitivity of asphalt.

## 1. Introduction

The transportation industry is growing day by day. Asphalt pavement plays an extremely important role in the modern transportation industry. Its excellent construction performance, driving comfort, and durability make it an indispensable part of modern transportation infrastructure construction [1,2]. However, the performance of asphalt is significantly affected by the ambient temperature. When the temperature changes, its physical and mechanical properties will undergo significant changes [3]. In high-temperature environments, the viscosity of asphalt decreases, and the hardness of asphalt mixtures drops, making road surfaces prone to deformation phenomena such as rutting and bulges. In low-temperature environments, the brittleness of asphalt increases, making asphalt mixtures more prone to fracture and thus causing cracking. During its service life, asphalt pavement undergoes repeated temperature changes. Such temperature cycles accelerate the aging process and the risk of rutting and cracking on asphalt pavement [4,5].

To reduce the effects of temperature changes on asphalt pavement, researchers have proposed many methods to address this challenge, including evaporative cooling, reflective cooling, thermochromic temperature regulation, etc. However, all the above-mentioned methods have their own drawbacks. For instance, evaporative cooling requires that the road surface maintain sufficient moisture. The reflection cooling method is greatly affected by the season, and the reflection cooling effect is influenced by the color of the aggregates. Thermochromic temperature regulation is costly, and it is difficult to balance the color-changing performance of the material, the wear resistance of the road surface, etc. Therefore, some scholars have proposed introducing PCMs into asphalt pavements [6,7,8,9].

PCMs have been widely applied in multiple fields at present, including aerospace, construction, clothing, energy, environmental protection, military fields, etc. [10,11,12,13]. There are various classification methods for phase change materials. The more commonly used classification method is to divide phase change materials into four types based on their phase state changes: solid–solid, solid–liquid, solid–gas, and liquid–gas [14]. Solid–solid PCMs have a small volume of phase change and no volatility or leakage. They have a relatively small impact on the mechanical properties of an asphalt mixture after being directly added to it. However, the enthalpy value of phase change is relatively low, and the temperature regulation effect is not good. Solid–gas and liquid–gas PCMs will produce gas after a phase change, with a large volume change, and are not suitable for practical engineering. Solid–liquid PCMs have been widely applied due to their high latent heat of phase change and small volume change [15,16,17]. Solid–liquid PCMs can be classified into inorganic PCMs and organic PCMs based on their chemical properties. Inorganic PCMs mainly include hydrate salts, metals, etc. [18] The thermal conductivity of hydrated salts is relatively low, with the thermal conductivity of most hydrated salts being around 0.5 W/(m·K). The low thermal conductivity limits the efficiency of heat energy transmission in hydrated salts [19]. Moreover, during the cooling process, hydrated salts are prone to supercooling and phase separation. This will reduce the release and absorption efficiency of thermal energy from hydrated salts. The supercooling and phase separation phenomena are usually suppressed by two methods: compounding with porous materials or microencapsulation.

Organic PCMs mainly include fatty acids, esters, paraffins, organic alcohols, polymer phase change materials, etc. [20]. Among them, fatty acids are fatty acid molecules composed of long-chain hydrocarbons. Common fatty-acid-based PCMs include decanoic acid, stearic acid, palmitic acid, lauric acid, etc., but they usually have relatively low thermal conductivity. Paraffin-based PCMs are organic PCMs based on paraffin, composed of a mixture of straight-chain alkanes with different numbers of carbon atoms. Their phase transition temperatures and enthalpy values can be adjusted by the composition of paraffin. Common paraffin-based phase change materials include paraffin, cetane, octadecane, etc. Tian et al. [21] used paraffin as a PCM and combined it with EG to prepare paraffin/EG CPCMs. Their experimental results proved that the enthalpy value of the phase change of the CPCM is close to that of paraffin, and no chemical reaction occurred between the two. Moreover, compared with paraffin, the heat transfer efficiency of the phase change material after compounding was significantly improved. Amini [22] et al. added copper oxide nano-powder (nano-CuO) as a PCM to an asphalt mixture, which reduced its temperature sensitivity. Cheng et al. [23] prepared PPGC-PCM by the graft copolymerization method and investigated the influence of PPGC-PCM on the properties of different asphalt mixtures.

To sum up, certain breakthroughs have been made in the research on self-regulating asphalt pavement. However, there are still some problems that restrict the development of critical temperature-regulating asphalt. At present, the critical temperature regulation technology for asphalt pavement only focuses on temperature changes in the pavement in high/low-temperature environments. There is no research on the dual-temperature domain pavement temperature regulation effect of simultaneously delaying the pavement temperature rise/drop in high/low-temperature environments. However, the area east of 27° N to 40° N latitude and 104° E longitude in our country does not apply to the regions that are hot in summer and cold in winter, which account for about 30% of China’s land area. In addition, most PCMs with relatively high enthalpy values have poor thermal conductivity by themselves, which weakens their temperature regulation ability. Therefore, this paper aims to develop a dual-PCM with both high and low phase transition temperatures and high thermal conductivity. This will enhance the performance and safety of the road surface.

## 2. Experiment and Materials

### 2.1. Raw Materials

Paraffin (PW) was selected as the PCM in this study. According to the temperature regulation range requirements of asphalt pavement, paraffin-5 (PW-5) was selected as the low-temperature PCM. Paraffin-44h (PW-44H) was used as the high-temperature PCM. The enthalpy values of the two materials are shown in Table 1. The mesh size of the selected expanded vermiculite (EVM) was 20 mesh to 40 mesh. The purity of nano-titanium dioxide (TiO_2_) was 99.8%.

### 2.2. Preparation of Composite PCMs and Double PCMs

**Figure 1:**
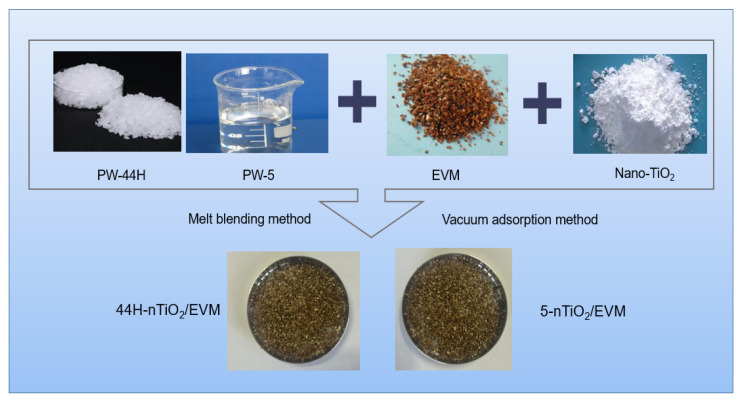
Preparation of high/low-temperature PCMs.

The preparation process of high-temperature composite PCMs was as follows (Figure 1). An appropriate amount of PW-44H was weighed, and a certain mass of EVM was dried in an oven at 120 °C until a constant weight was achieved. PW-44H was heated at 80 °C and melted into a liquid state. Then, a certain mass of nano-TiO_2_ was weighed. The three were mixed in a certain proportion and placed in the same beaker. The mixture was stirred in an oil bath pot at a temperature of 80 °C using a high-torque electric stirring rod. The stirring rate was 1000 r/min and the stirring duration was 20 min to prepare 44H-nTiO_2_/EVM high-temperature CPCM.

The preparation process of low-temperature CPCMs was as follows. An appropriate amount of PW-5 was weighed, and a certain mass of EVM was dried in an oven at 120 °C until a constant weight was achieved. A certain mass of nano-TiO_2_ was weighed. The three were mixed a certain proportion and placed in the same beaker. The 5-nTiO_2_/EVM low-temperature CPCM was prepared by stirring with a high-torque electric stirring rod at a stirring rate of 1000 r/min and a stirring duration of 20 min.

The high- and low-temperature CPCMs were compounded in a 1:1 ratio to obtain the double phase change composite materials.

### 2.3. Preparation of Double PCM Asphalt

Double PCM asphalt was prepared by directly mixing double phase change materials into the original asphalt in mass ratios of 10%, 20%, and 30%. According to the previous achievements of the research group [24], the original asphalt was heated in an oven at 165 °C ± 5 °C until it reached a flowing state. Then, it was transferred into a 170 °C constant-temperature oil bath pot, and the prepared double phase change material was slowly added. The mixture was stirred with a glass rod for about 10 min to dissolve the double phase change material into the asphalt; then, the mixture was subjected to 20 min of vigorous stirring (500 rpm) in a high-torque electric mixer. In the end, the mixture was stirred with a rod for 10 min to remove the bubbles in the double PCM asphalt, and the double PCM asphalt sample was thus prepared.

### 2.4. Testing and Characterization

#### 2.4.1. Heat Storage Performance

The heat storage performance of the double CPCMs was tested by DSC. The test range for high-temperature PCMs was from 25 °C to 60 °C. The test temperature range for low-temperature PCMs was from −15 °C to 15 °C. Moreover, the temperature change rates of both materials were set at 2 °C/min. Each sample was placed in the aluminum alloy closed sample tray of the DSC and heated and cooled. The heat flow signal and temperature change curve were obtained by the DSC instrument.

The heat storage performance of the original sample asphalt and the double phase change asphalt was investigated by DSC. The testing process was divided into two parts: the heating stage and the cooling stage. When cooling, the temperature was set from 10 °C to −10 °C; when heating, the temperature was set from 25 °C to 60 °C, and the testing process was the same as above.

#### 2.4.2. Thermal Conductivity

The thermal conductivity of the CPCMs was tested by using a HotDisk TPS 3500 (Swedish HOTDISK Company, Gothenburg, Sweden) testing instrument in accordance with the international standard ISO 22007-2 [25]. This testing instrument adopts the principle of Transient Plane Source (TPS) and is capable of accurately measuring the thermophysical properties of solid materials. It is especially suitable for determining the thermal conductivity of samples. First, a sample of the appropriate size for testing was cut and placed on the test platform of the HotDisk TPS 3500. During the testing process, the instrument applied a transient thermal pulse to the sample surface through a heat source probe. Due to the transmission of thermal pulses, temperature changes occurred inside the sample, and this process was perceived by the probes and recorded in real time. By measuring the variation in the surface temperature of the sample over time, combined with the known geometric parameters and physical models, the thermal conductivity was calculated using the algorithm in the ISO 22007-2 standard.

#### 2.4.3. Microscopic Properties

The microscopic properties of the composite PCM were analyzed by SEM. A sample of the appropriate size was selected and stuck onto the conductive adhesive of the SEM sample stage, trying to keep the sample surface as flat as possible. The samples were gold-sprayed to ensure that the electron beam could scan and image stably during the SEM testing process. Then, the processed samples were placed on the test bench. The working voltage and scanning conditions of the scanning electron microscope were adjusted, and the microstructure characteristics, including particle morphology, distribution, porosity, and other surface features, were observed. Finally, the microscopic morphological characteristics of the CPCMs were evaluated.

#### 2.4.4. Thermal Stability

The thermal stability of the CPCM was tested by using a differential thermal comprehensive analyzer (TG-DTA, model: Labsys Evo) (Setalam Company of France, Antipolis, France). The temperature range for the test was 25–450 °C, and the heating rate was 10 °C/min. An appropriate amount of the sample was placed in an aluminum sample tray, which was placed in the high-temperature furnace of the instrument. At the beginning of the experiment, the mass of the sample was recorded at room temperature. Then, the temperature was gradually increased, the mass change of the sample was monitored, and the TG curve was drawn. The mass change of the CPCM at different temperatures was analyzed through the TG.

#### 2.4.5. Phase Change Durability

In order to study the phase change durability of the CPCMs during application, phase change cycle experiments of heating and cooling were conducted on the CPCMs. An appropriate amount of high/low-temperature composite PCMs was weighed and placed on dry weighing paper. Then, the weighing paper was placed in the environmental box. The maximum temperature of the environmental chamber was set to 75 °C, and the minimum temperature was set to −15 °C. Multiple heating/cooling cycles between the highest and lowest temperatures were performed. When the number of cycles was 20 and 50, respectively, the weighing paper was taken out, and the mass of the high/low-temperature composite PCMs remaining on the weighing paper was determined. The remaining mass of the material was calculated after 20 and 50 phase transformation cycles.

#### 2.4.6. Rheological Properties of Double Phase Change Asphalt

The high-temperature rutting resistance of the original asphalt and the double PCM asphalt was evaluated by using the rutting factor. The test was conducted using the temperature scanning mode, with the experimental frequency set at 10 rad/s, the test temperature range set at 46–76 °C, and the temperature test interval set at 6 °C. A higher rutting factor usually indicates that the asphalt material has a stronger resistance to rutting.

(1) $$Rutting\;factor=\frac{G^{*}}{\sin\delta }$$

where G* is the dynamic shear complex modulus of asphalt and δ is the phase angle of asphalt.

The medium-temperature fatigue resistance of the original asphalt and the double phase change asphalt was evaluated by using the G-R parameter as the index. The experiment was conducted in frequency scanning mode, with the temperature set at 5 °C, 15 °C, and 25 °C and the strain magnitude set at 1%. A lower G-R parameter indicates that the asphalt material can maintain good elastic recovery under stress and has good fatigue resistance. The specific calculation formula is as follows:

(2) $$G-R=\frac{G^{*}\cdot {\left(\cos\delta \right)}^{2}}{\sin\delta }$$

where G* is the dynamic shear complex modulus of asphalt at 15 °C and 0.005 rad/s and δ is the phase angle of asphalt at 15 °C and 0.005 rad/s.

The low-temperature crack resistance of the original asphalt and the double phase change asphalt was evaluated by using the creep modulus S and creep rate m. The experiment was conducted in frequency scanning mode, with the frequency set at 0.2 to 100 rad/s, the temperature set at −6 °C, −12 °C, and −18 °C, and the strain magnitude set at 1%. The creep modulus S reflects the anti-deformation capacity of asphalt. The higher the S value, the stronger the rigidity of asphalt. The smaller the m value, the slower the creep rate of asphalt, and it can better maintain its shape at low temperatures.

#### 2.4.7. Temperature Regulation Performance of Double PCM Asphalt

The influence of the dosage on the temperature regulation performance of double PCMs in asphalt was evaluated. The same mass of the original sample asphalt and the double PCM asphalt with dosages of 10%, 20%, and 30% was weighed. All the samples to be tested were placed in the environmental chamber, and a PT100 platinum resistance sensor (Yuyao Tenghui Temperature Control Instrument Factory, Yuyao, China) and a temperature recorder were used to detect the temperature changes in the samples. The temperature probe was placed at the center of each asphalt sample. When testing the temperature regulation performance within the high-temperature range, the temperature change of the environmental chamber was set to 25–65 °C. When testing the temperature regulation performance within the low-temperature range, the temperature variation of the environmental chamber was set at −20 °C to −20 °C. The calculation method of the heating/cooling rate is as follows:

(3) $$V_{1}=\frac{T_{h}-T_{l}}{\Delta t}$$

where V_1_ L is the heating rate of the sample, with the unit being °C/min. T_h_ is the highest temperature of the sample during the heating process, with the unit being °C. T_l_ is the lowest temperature of the sample during the heating process, with the unit being °C.

(4) $$V_{2}=\frac{T_{h}-T_{l}}{\Delta t}$$

where V_2_ is the cooling rate of the sample, with the unit being °C/min. T_h_ is the highest temperature of the sample during the heating process, with the unit being °C. T_l_ is the lowest temperature of the sample during the heating process, with the unit being °C.

## 3. Results and Discussion

### 3.1. Research on the Adsorption Ratio of PCMs

**Figure 2:**
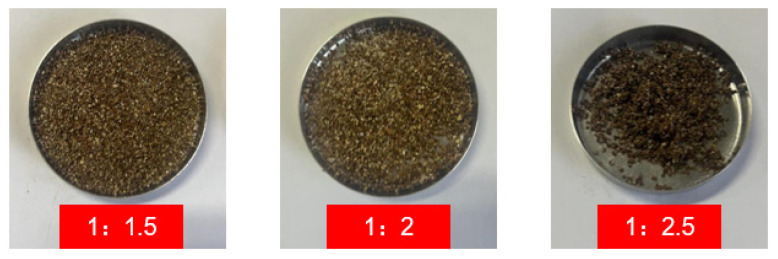
Different mass ratios of 44H/EVM CPCMs.

We determined the optimal adsorption ratio of paraffin as the PCM and expanded vermiculite as the carrier matrix in the composite PCM. In this paper, samples with mass ratios of EVM to PW-44H of 1:1.5, 1:2, and 1:2.5 were prepared. After going through the same preparation steps, the macroscopic morphology of the prepared CPCM is shown in Figure 2.

Figure 1 shows that when the mass ratio of PW-44H to EVM is 2.5:1, severe agglomeration occurs in the CPCM. The EVM particles are stuck together, indicating that at this ratio, EVM can no longer completely adsorb PW, and some PW still remains on the surface of EVM. After the temperature drops, the phase turns solid, causing the EVM particles to stick to each other and be unable to disperse. When the mass ratio of PW-44H to EVM is 1.5:1 and 2:1, no obvious clumping phenomenon occurs. Overall, it is rather loose. It is indicated that under both of these proportions, EVM can adsorb the added PW without any obvious leakage problem. In order to increase the overall phase change enthalpy value of the CPCM and enhance the temperature regulation effect of the CPCM, ultimately, 2:1 was chosen as the adsorption ratio.

### 3.2. Research on the Dosage of Nano-TiO_2_

**Figure 3:**
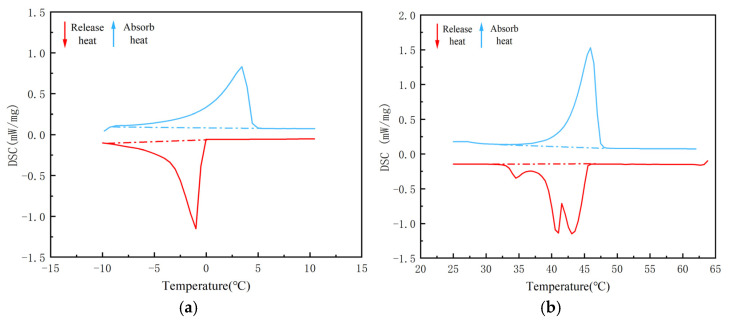
DSC curves of the CPCM without nano-TiO_2_ added: (**a**) 5/EVM composite phase change material; (**b**) 44H/EVM composite PCM.

**Figure 4:**
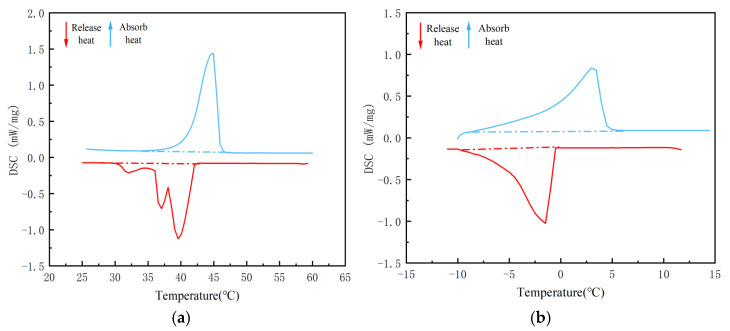

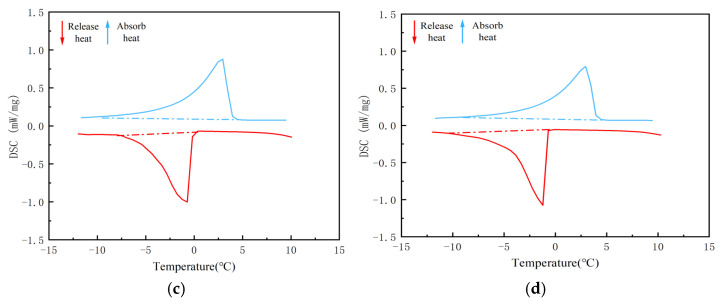
DSC curves of the low-temperature composite PCM with nano-TiO_2_ added: (**a**) 1%, (**b**) 2%, (**c**) 3%, and (**d**) 4%.

**Figure 5:**
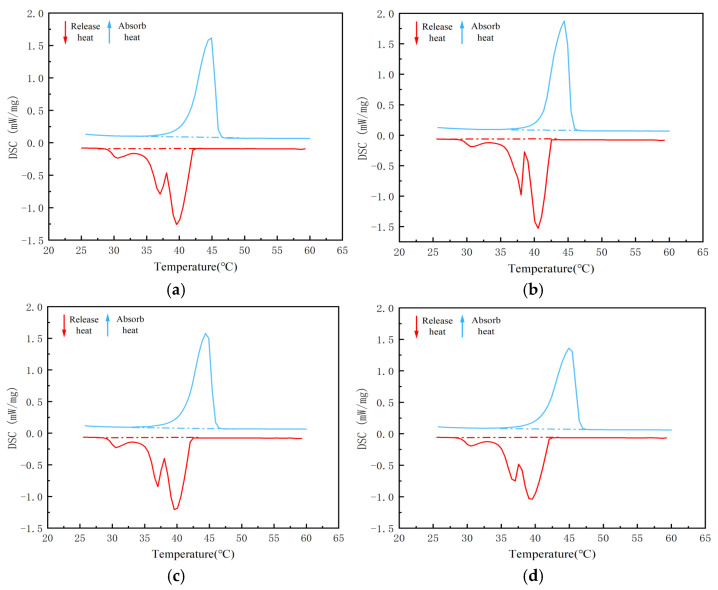
DSC curves of the high-temperature CPCM with nano-TiO_2_ added: (**a**) 1%, (**b**) 2%, (**c**) 3%, and (**d**) 4%.

Due to the poor thermal conductivity of PW and EVM, we attempted to enhance the overall thermal conductivity of the composite PCM and improve the efficiency of heat transfer between the composite PCM and asphalt. In this paper, nano-TiO_2_ was selected as the thermal conductor and added to the CPCM to prepare the CPCM. However, when the dosage of nano-TiO_2_ is too low, the improvement in the thermal conductivity of the CPCM is insufficient; when the dosage is too high, it tends to affect the adsorption of the PCM. Therefore, it is necessary to confirm the optimal dosage of nano-TiO_2_ in the CPCM. In phase change materials, the dosage of nano-TiO_2_ is generally within the range of 1% to 4% [24]. Therefore, in this paper, taking the quality of the carrier matrix as the benchmark, CPCMs without nano-TiO_2_ added and those with a nano-TiO_2_ content of 1%, 2%, 3%, and 4% were prepared. Figure 3 shows the DSC curve of the CPCM prepared without adding nano-TiO_2_. Figure 4 and Figure 5 show the DSC curves of the CPCMs prepared by adding nano-TiO_2_. Table 1 and Table 2 show the phase transition temperature and enthalpy of the CPCMs. The enthalpy of phase change data was calculated from the peak area of the DSC curve.

Table 3 lists the results when the dosage of nano-TiO_2_ is 1–2%. With the increase in its dosage, the phase transformation temperature range of the CPCM is gradually shortened. Compared with before the addition of nano-TiO_2_, the field of the phase transition temperature became more concentrated. During the cooling stage, compared with 5/EVM, the phase transition temperature range of 5/EVM-1 hardly changed. The phase transition range of 5/EVM-2 was shortened by 12.6%, the phase transition temperature range of 5/EVM-3 was shortened by 11.4%, and the phase transition temperature range of 5/EVM-4 was extended by 5.2%. During the heating stage, compared with 44H/EVM, the phase transition temperature range of 44H/EVM-1 was shortened by 20%, that of 44H/EVM-2 was shortened by 38.8%, that of 44H/EVM-3 was shortened by 18.5%, and that of 44H/EVM-4 remained almost unchanged. When the dosage of nano-TiO_2_ is 1–3%, the enthalpy phase change values of the CPCMs do not change significantly. However, when the dosage reached 4%, the enthalpy value of the CPCM decreased significantly. The experimental results show that within a certain range, the incorporation of nano-TiO_2_ can increase the thermal conductivity of the CPCM and make the phase change range of the composite PCM more concentrated. However, an excessively high dosage will instead reduce the enthalpy value of the CPCM, affecting the adsorption capacity of expanded vermiculite for paraffin. Therefore, 2% is selected as the final dosage of nano-TiO_2_.

### 3.3. Analysis of Thermal Conductivity of the CPCMs

**Figure 6:**
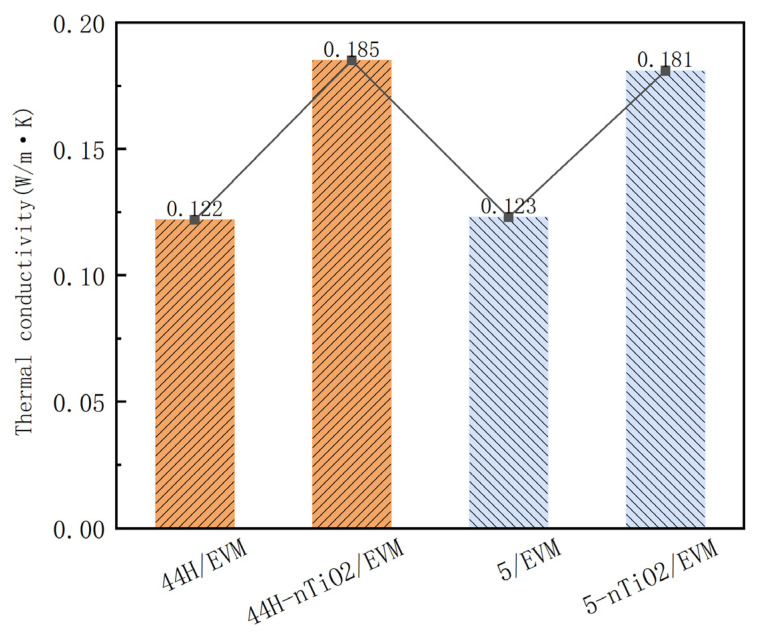
Thermal conductivity of CPCMs.

The thermal conductivity of the CPCMs before and after the addition of nano-TiO_2_ was detected by using the transient plane source method. Figure 6 shows the thermal conductivity coefficients of four CPCMs at room temperature.

As shown in Figure 6, before the addition of nano-TiO_2_, the thermal conductivity of the high-temperature CPCM was 0.122 W/(m·K), and that of the low-temperature CPCM was 0.123 W/(m·K). After incorporating nano-TiO_2_, the thermal conductivity of the high-temperature CPCM was 0.185 W/(m·K), and that of the low-temperature CPCM was 0.181 W/(m·K). The growth rates of thermal conductivity were 51.6% and 47.2%, respectively. The efficiency of heat transfer between the CPCM and asphalt can be improved by adding nano-TiO_2_. The reason lies in the fact that the inherent high thermal conductivity of nano-TiO_2_ can form continuous or semi-continuous heat conduction paths in the CPCM matrix, significantly reducing thermal resistance. When a PCM is added to asphalt, these heat conduction networks can promote the rapid heat transfer in the asphalt matrix and reduce the local temperature gradient. Meanwhile, the hydroxyl groups (-OH) of nano-TiO_2_ may interact with the polar components in asphalt (such as asphaltenes), forming a stronger interfacial bond, thereby reducing the interfacial thermal resistance between PCM and asphalt.

### 3.4. Microscopic Morphology Analysis

**Figure 7:**
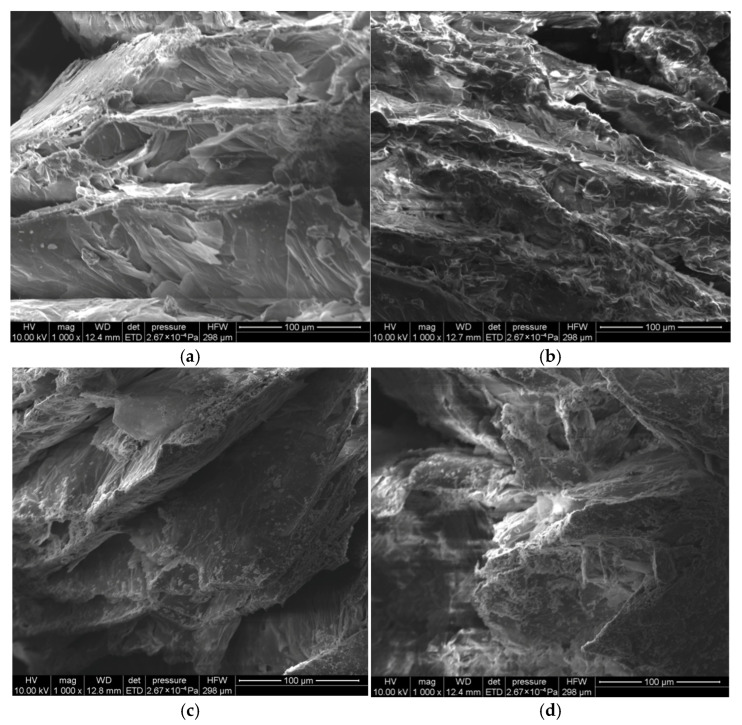
Microscopic morphology of CPCMs: (**a**) 5/EVM, (**b**) 44H/EVM, (**c**) 5-nTiO2/EVM, and (**d**) 44H-nTiO2/EVM.

Figure 7 shows the SEM microscopic morphology of the CPCM. According to Figure 7a–d, a large number of pores in the CPCM were filled with paraffin under the action of vacuum pressure. Moreover, under the action of capillary force and van der Waals force, paraffin was effectively fixed in the pores of expanded vermiculite. The addition of nano-TiO_2_ had no effect on the adsorption of paraffin, and there was no significant difference in the SEM morphology of the CPCM before and after the addition.

### 3.5. Thermal Stability Analysis

**Figure 8:**
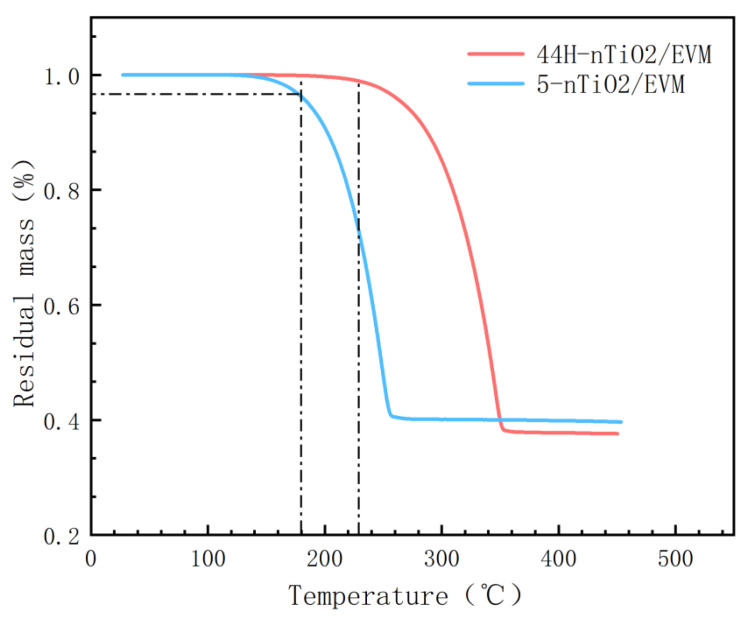
Thermogravimetric curve of the composite PCM.

To explore the thermal stability of CPCMs, TG was used to test the thermal stability of the composite PCMs within the temperature range of 25 °C to 450 °C. In Figure 6, the decomposition initial temperature of the low-temperature PCM 5-nTiO_2_/EVM is relatively low. However, the high-temperature composite PCM 44H-nTiO_2_/EVM shows a relatively high decomposition initiation temperature. At 180 °C, the remaining masses of 5-nTiO_2_/EVM and 44H-nTiO_2_/EVM were 96.2% and 99.9% respectively. The corresponding mass losses were 3.8% and 0.1% respectively, indicating that these two materials have good thermal stability at high temperatures. As the temperature rose further, the two materials began to undergo decomposition to varying degrees. Moreover, the decomposition rate of 5-nTiO_2_/EVM was significantly faster than that of 44H-nTiO_2_/EVM. Specifically, 44H-nTiO_2_/EVM began to decompose only at 225 °C, and the decomposition rate was relatively slow. However, when the temperature rose to 285 °C, its decomposition rate accelerated. The residual mass of the material tended to stabilize at 365 °C. The final residual mass of the two materials was basically consistent with the relative mass of the PCM and the carrier matrix. Figure 8 shows the thermogravimetric curve of the modified composite phase change material.

### 3.6. Phase Change Durability Analysis

To study the phase change durability of CPCMs, thermal cycling experiments were conducted on them [26]. The data in Table 4 illustrate that after multiple heating and cooling phase change cycles from −15 °C to 75 °C, the masses of both composite phase change materials decreased in a small range. This indicates that some core materials have experienced leakage. When the temperature cycle is 20 times, the mass loss of the high/low-temperature CPCMs is 6% and 5%, respectively. However, when the number of phase change cycles reaches 50, compared with 20, the remaining mass of the CPCMs only decreases by 1%. Moreover, the enthalpy value of the phase transition did not change significantly either. The possible reason is that during the first 20 phase change cycles, part of the core material attached to the surface of the carrier matrix was heated and released from the adsorption force, thus causing leakage during the experiment. However, after 50 cycles, the two composite phase change materials still maintained over 93% of their pre-cycle mass and phase change enthalpy values, indicating that the CPCMs have excellent phase change durability.

### 3.7. Analysis of Thermal Storage Performance of Double PCM Asphalt

**Figure 9:**
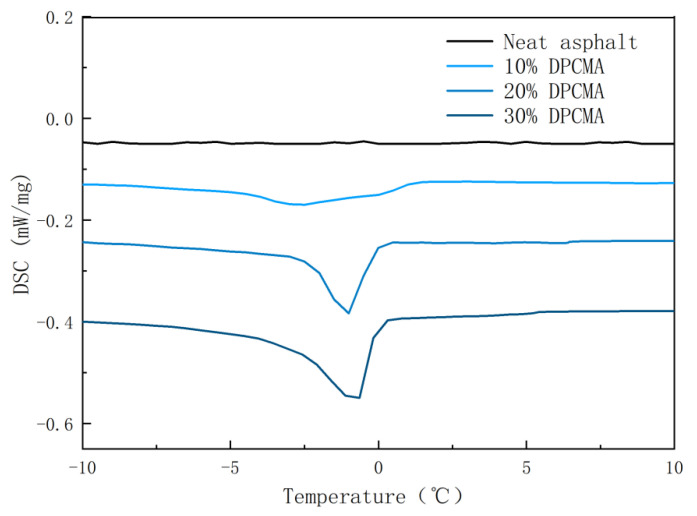
The exothermic DSC curves of the double phase change asphalt under different dosages of double phase change materials.

**Figure 10:**
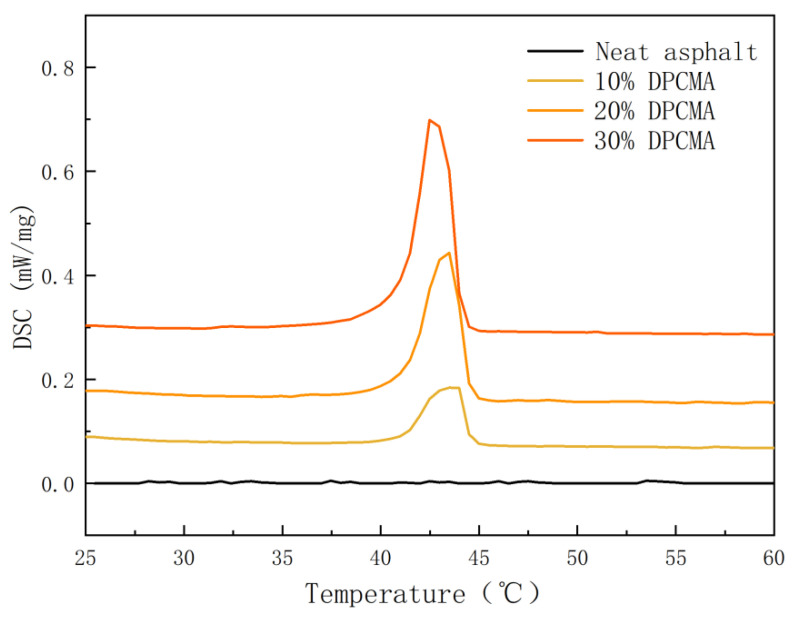
The heat absorption DSC curves of the double phase change asphalt under different dosages of double phase change materials.

The high- and low-temperature CPCMs prepared in the previous section were compounded in a 1:1 ratio to obtain double phase change composite materials. Then, they were added to asphalt at dosages of 10%, 20%, and 30% to obtain double phase change asphalt. To explore whether phase change materials can still undergo a phase change to absorb or release heat after the addition of asphalt, DSC experiments were conducted on the double PCM asphalt. Figure 9 and Figure 10 show the results of the double phase change asphalt. Table 5 shows the DSC results of double-PCM asphalt.

It can be seen from Figure 9 and Figure 10 that when the dosage of double PCM is 0%, the DSC curve is basically a straight line, indicating that the original asphalt cannot undergo a phase change. When the double phase change asphalt with added double PCMs shows obvious peaks, it indicates that the double PCMs play a phase change role in the double phase change asphalt. The results indicate that the incorporation of double PCMs can endow double phase change asphalt with the ability to self-regulate temperature through heat absorption and heat release.

### 3.8. Analysis of the Rheological Properties of the Double PCM Asphalt

#### 3.8.1. High-Temperature Performance Analysis

**Figure 11:**
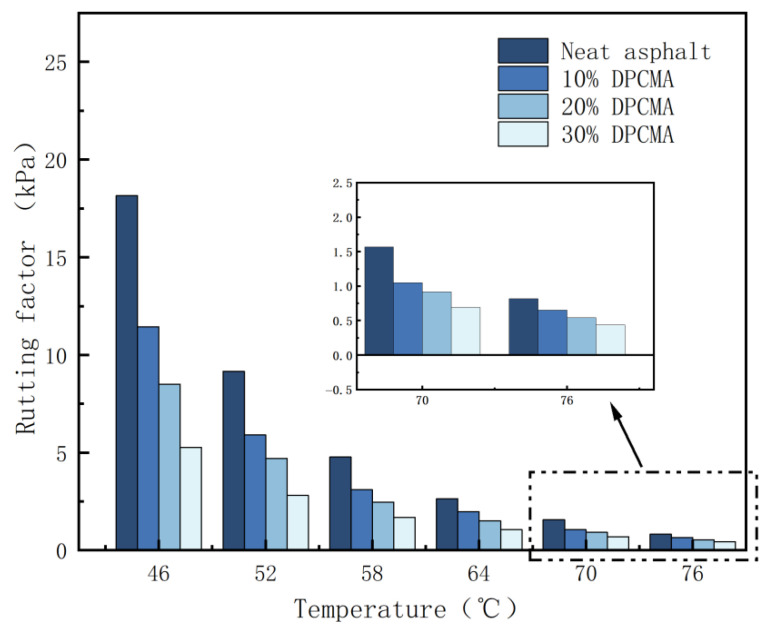
Rutting factors of the original asphalt and the double phase change asphalt.

Figure 11 shows the rutting factors of the original asphalt and the double PCM asphalt. As the dosage of the double PCMs increases, the rutting factor of the double PCM asphalt shows a downward trend. This might be due to the fact that at high temperatures, part of the paraffin phase turns into liquid and overflows from the expanded vermiculite, mixing with the asphalt and reducing the elastic modulus of the asphalt. This leads to a reduction in the rutting factor of the double PCM asphalt.

#### 3.8.2. Analysis of Fatigue Resistance Performance

**Figure 12:**
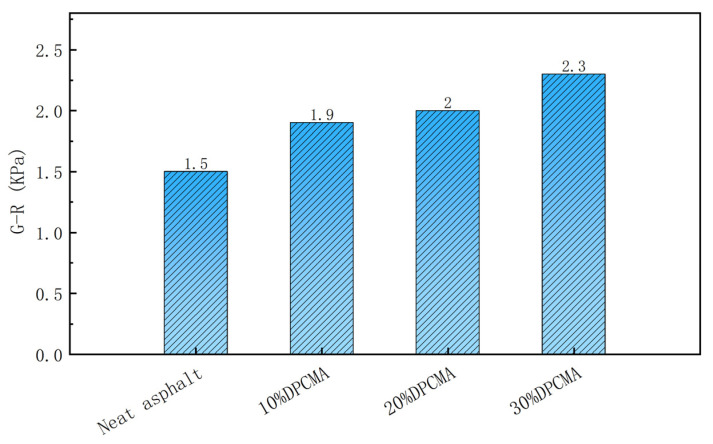
G-R parameters of the double PCM asphalt under different dosages of double PCMs.

The fatigue resistance performance of the double phase change asphalt was evaluated using the G-R parameter [24]. Figure 12 shows the G-R parameters results.

With the increase in the dosage of the double PCMs, the G-R parameter of the double phase change asphalt also shows an upward trend, but the increasing trend is not obvious. Compared with the original asphalt, the increase in the G-R parameters of the double PCM asphalt was 0.4 KPa, 0.5 KPa, and 0.8 KPa, respectively, and the growth rates were 26.7%, 33.3%, and 53.3%, respectively. It can be found that the incorporation of double PCMs does not have an impact on the medium-temperature fatigue resistance of the original asphalt. The reason for the increase in the G-R parameter might be that within the test temperature range, the low-temperature PCM PW-5 is in a molten state, and its viscous flow absorbs part of the mechanical energy. However, it may reduce the elastic recovery ability (increase the δ angle), resulting in a slight increase in the G-R parameter. Overall, it is still far lower than the initial cracking value of 180 KPa; thus, it still has relatively good fatigue resistance.

#### 3.8.3. Analysis of Low-Temperature Crack Resistance Performance

**Figure 13:**
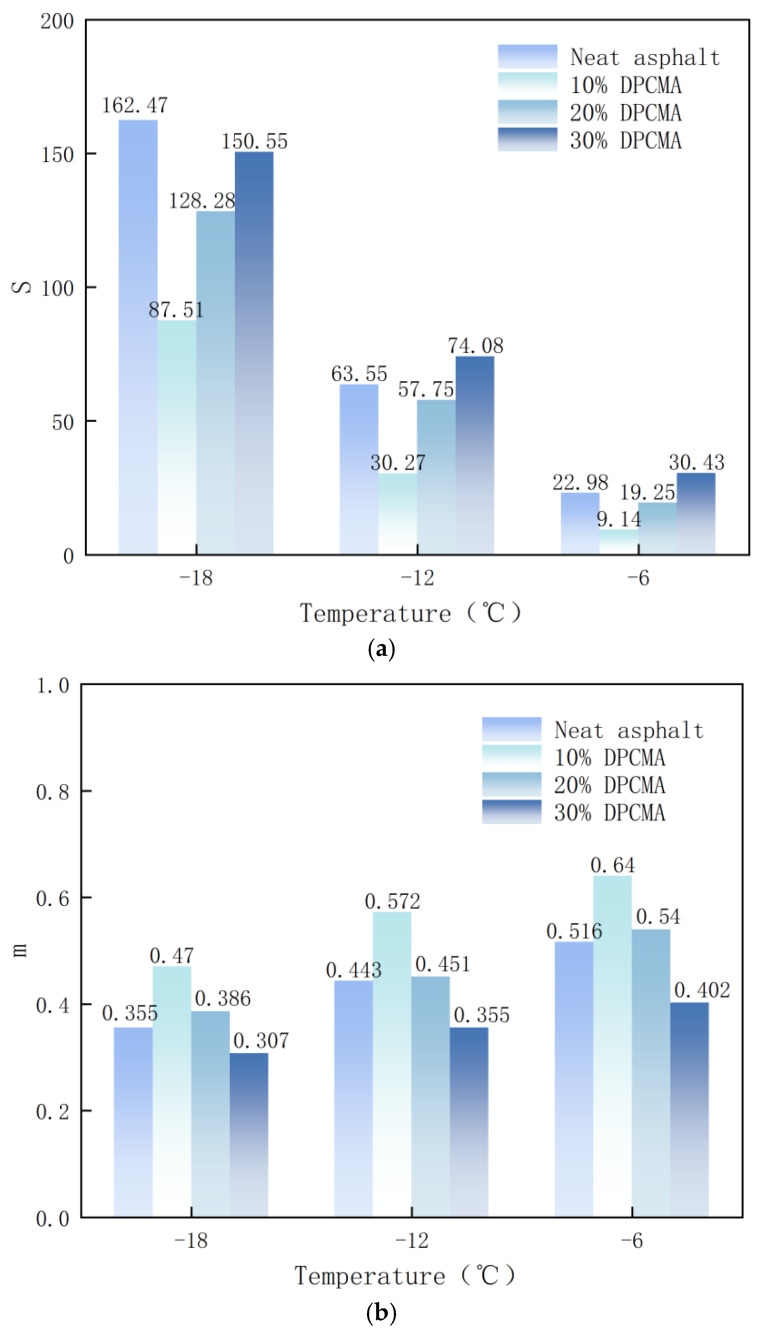
Low-temperature performance of the double PCM asphalt under different dosages of double PCMs. (**a**) The stiffness modulus of the double phase change asphalt under different dosages of double PCMs. (**b**) The creep rate of the double PCM asphalt under different dosages of double PCMs.

Figure 13 shows the low-temperature performance of the double PCM asphalt at different dosages.

It is indicated that as the dosage increases, the asphalt gradually hardens, and its low-temperature deformation capacity also gradually decreases. This is because the hardness of PCMs is stronger than that of the original asphalt. After being mixed with asphalt, the overall hardness of the double phase change asphalt increases. Therefore, when the dosage increases, the overall hardness of the double phase change asphalt will also increase. However, compared with the original asphalt, at −18 °C, the S values of the three dosages are all smaller than those of the original asphalt. At −12 °C and −6 °C, the S value of the 10% double phase change asphalt is less than that of the original sample asphalt; the value of the 20% double phase change asphalt is close to that of the original sample asphalt; and the S value of the 30% double phase change asphalt is greater. As shown in Figure 13b, with the increase in the dosage of the double PCM, the creep rate m of the double phase change asphalt will decrease accordingly. It is indicated that as the dosage increases, the relaxation capacity of the double phase change asphalt decreases, and the possibility of cracking at low temperatures is higher. Compared with the original asphalt, the m value of the 10% content double phase change asphalt is larger, while the m value of the 30% content double phase change asphalt is smaller. The reason might be that in a low-temperature environment, the crystallization behavior of PW-5 releases heat, delays the glass transition of asphalt, and increases the creep rate m value. However, as the dosage of the double PCM increases, the dosage of rigid particles in the double phase change asphalt also increases, offsetting this effect.

### 3.9. Analysis of the Temperature Regulation Performance of the Double PCM Asphalt

**Figure 14:**
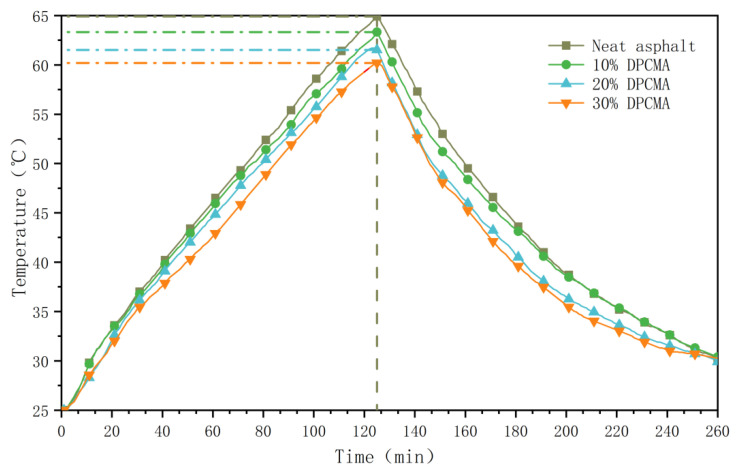
Temperature rise curves of the original asphalt and the double PCM asphalt.

**Figure 15:**
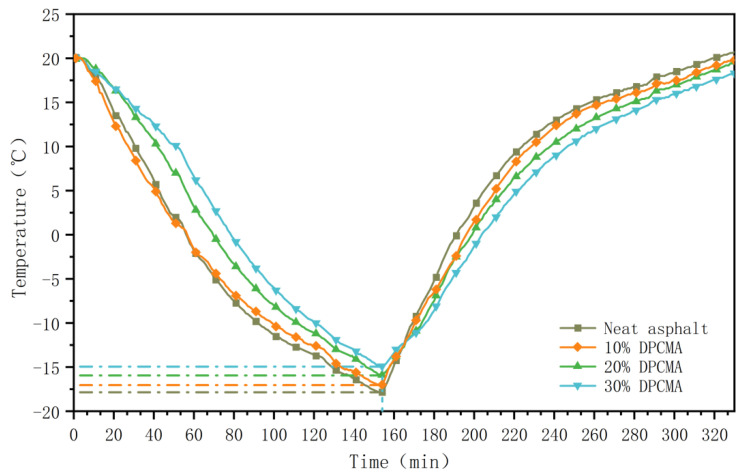
Cooling temperature curves of the original asphalt and the double phase change asphalt.

The temperature changes in the double phase change asphalt were tested in an environmental chamber. The results are shown in Figure 14 and Figure 15.

As the data in the above figure show, with the increase in the dosage of the double PCM, the rate of heating and cooling also significantly decreases. In the temperature rise curve, when the temperature rises to 64.9 °C, the cooling amplitudes of the double phase change asphalt with dosages of 10%, 20%, and 30% are 1.5 °C, 3.4 °C, and 4.7 °C, respectively. When the dosage is 20%, the heating rate of the original asphalt decreases from 0.319 °C/min to 0.292 °C/min, representing a reduction of 8.5%. In the cooling temperature curve, when the temperature of asphalt without PCMs drops to 17.9 °C, the temperature increase amplitudes of the double phase change asphalt with dosages of 10%, 20%, and 30% are 0.8 °C, 2.1 °C, and 3.0 °C, respectively. When the dosage is 20%, the cooling rate of the original asphalt decreases from 0.248 °C/min to 0.234 °C/min, representing a reduction of 5.6%. From the above comparison, it can be seen that the double PCM has a good temperature regulation effect in both high- and low-temperature ranges. The synergistic effect of it in high- and low-temperature environments enables asphalt to have an adaptive temperature regulation ability within a wide temperature range.

## 4. Conclusions

In this paper, paraffin wax, expanded vermiculite, and nano-TiO_2_ are used as raw materials. The 44H-nTiO_2_/EVM and 5-nTiO_2_/EVM CPCMs were prepared by combining the melt blending method with the vacuum adsorption method. Two CPCMs were compounded in a 1:1 ratio to prepare a double PCM. Its performance was investigated by DSC, SEM, and TG. Subsequently, the double PCMs were added to the asphalt at ratios of 10%, 20%, and 30%, and the performance of the double PCM asphalt was characterized by DSC and DSR.

(1) When the dosage of nano-TiO_2_ is 2%, it can effectively increase the thermal conductivity of the composite PCM. The thermal conductivity growth rates of the high- and low-temperature composite PCMs are 51.6% and 47.2%, respectively. In addition, the addition of nano-TiO_2_ makes the phase change temperature range of the CPCMs more concentrated and does not affect the adsorption and phase change enthalpy values of the CPCMs. The phase transition enthalpy values of the high/low-temperature CPCMs 5-nTiO_2_/EVM and 44H-nTiO_2_/EVM are 106.6 J/g and 150.8 J/g, respectively. Moreover, at a high temperature of 180 °C, the remaining masses of the two are 96.2% and 99.9%, respectively.
(2) With the increase in the dosage of double PCM, the temperature regulation performance of the double phase change asphalt also improves accordingly. When the dosage of the double PCM is 10%, 20%, and 30%, it can reduce the high-temperature extreme value of asphalt by 1.5 °C, 3.4 °C, and 4.7 °C and increase the low-temperature extreme value by 0.8 °C, 2.1 °C, and 3.0 °C. When the dosage of the double PCM is 20%, compared with the original asphalt, the heating rate of the double PCM decreases by 8.5%, and the cooling rate decreases by 5.6%.
(3) The high-temperature and medium-temperature performance of the double PCM asphalt is somewhat lower than that of the original asphalt, but the influence on the medium-temperature fatigue resistance is not significant. In terms of low-temperature crack resistance, the performance improves when the dosage is 10%, but it decreases when the dosage is 30%. When the dosage is 20%, the performance of the double PCM asphalt is closest to that of the original asphalt. Furthermore, when the dosage of the double PCM increases from 10% to 20%, the growth in the temperature regulation effect of the double PCM is greater than that when the dosage of the double PCM increases from 20% to 30%. Therefore, in order to enhance the temperature regulation effect while reducing the negative impact of the double PCM on the high-temperature performance of asphalt, and to ensure that the medium-temperature fatigue resistance and low-temperature crack resistance of the double phase change asphalt do not differ significantly from those of the original asphalt, the experimental results show that 20% is a relatively reasonable dosage of the double PCM.

## Figures and Tables

**Table 1:** Heat storage performance of PCMs.

Material Type	Phase Transition Temperature Range (°C)	Enthalpy of Phase Change (J/g)
PW-5	−8.4–1.0	153.0
PW-44H	38.8–46.4	249.1

**Table 2:** CPCMs without the addition of nano-TiO_2_.

Material Type	Heating Up				Cool Down			
	Initial Temperature (°C)	Peak Temperature (°C)	Termination Temperature (°C)	Enthalpy Value (°C)	Initial Temperature (°C)	Peak Temperature (°C)	Termination Temperature (°C)	Enthalpy Value (J/g)
5/EVM	−9.8	3.4	5.0	96.9	0	−1.0	−9.5	104.1
44H/EVM	37.1	47.9	50.5	156.4	44.0	41.0	30.5	160.1

**Table 3:** DSC test results of the CPCM with nano-TiO_2_ added.

Material Type	Heating Up				Cool Down			
	Initial Temperature (°C)	Peak Temperature (°C)	Termination Temperature (°C)	Enthalpy Value (J/g)	Initial Temperature (°C)	Peak Temperature (°C)	Termination Temperature (°C)	Enthalpy Value (J/g)
5/EVM-1	−8.7	2.9	4.4	100.7	0	−1.5	−9.4	103.7
5/EVM-2	−8.1	2.9	3.9	101.5	0.4	−0.7	−7.9	106.6
5/EVM-3	−9.1	3.0	4.1	100.0	0.5	−0.7	−7.9	103.9
5/EVM-4	−8.6	2.9	4.4	88.5	0	−1.2	−9.9	92.5
44H/EVM-1	36.1	44.9	46.9	149.5	43.0	39.5	34.0	155.6
44H/EVM-2	37.7	44.4	45.9	150.8	42.5	40.5	35.0	155.8
44H/EVM-3	35.5	44.4	46.5	151.3	43.0	39.5	33.5	155.3
44H/EVM-4	34.4	44.9	47.4	138.1	42.5	39.5	33.0	142.2

**Table 4:** Phase change durability of the CPCMs.

Phase Change Material	After 20 Cycles		After 50 Cycles	
	Residual Mass (%)	Enthalpy of Phase Change (J/g)	Residual Mass (%)	Enthalpy of Phase Change (J/g)
44H-nTiO_2_/EVM	94	140.3	93	139.2
5-nTiO_2_/EVM	95	100.4	94	98.9

**Table 5:** DSC results of the double PCM asphalt.

Dosage of Double Phase Change Materials	Cool Down				Heating Up			
	Initial Temperature (°C)	Peak Temperature (°C)	Termination Temperature (°C)	Enthalpy Value (J/g)	Initial Temperature (°C)	Peak Temperature (°C)	Termination Temperature (°C)	Enthalpy Value (J/g)
0	-	-	-	-	-	-	-	-
10%	0	−2.5	−4.0	5.4	38.5	42.5	44.5	8.4
20%	0.5	−1.0	−3.5	8.2	39.4	43.5	44.9	18.8
30%	0.3	−0.6	−4.0	13.4	40.5	43.5	45.0	28.1

## Data Availability

The original contributions presented in this study are included in the article. Further inquiries can be directed to the corresponding author.

## References

[1] Guo M., Liang M., Jiao Y., Zhao W., Duan Y., Liu H. (2020). A review of phase change materials in asphalt binder and asphalt mixture. Constr. Build. Mater..

[2] Guo M., Zhang R., Du X., Liu P. (2024). A state-of-the-art review on the functionality of ultra-thin overlays towards a future low carbon road maintenance. Engineering.

[3] Chen Y., Wang H., You Z., Hossiney N. (2020). Application of phase change material in asphalt mixture—A review. Constr. Build. Mater..

[4] Zhao X., Shen A., Ma B. (2018). Temperature adaptability of asphalt pavement to high temperatures and significant temperature differences. Adv. Mater. Sci. Eng..

[5] Ding T., Wei S., Ren X., Guo M., Yu D. (2024). Multiscale evaluation on temperature adjusting performance and road performance of asphalt mixture containing dual phase change materials. Constr. Build. Mater..

[6] Manning B.J., Bender P.R., Cote S.A., Lewis R.A., Sakulich A.R., Mallick R.B. (2015). Assessing the feasibility of incorporating phase change material in hot mix asphalt. Sustain. Cities Soc..

[7] Farah S., Farouk F., Pascal H.B. (2016). Phase change materials (PCM) for cooling applications in buildings: A review. Energy Build..

[8] Athukorallage B., Dissanayaka T., Senadheera S., James D. (2018). Performance analysis of incorporating phase change materials in asphalt concrete pavements. Constr. Build. Mater..

[9] Kakar M.R., Refaa Z., Worlitschek J. (2018). Use of microencapsulated phase change materials in bitumen to mitigate the thermal distresses in asphalt pavements. RILEM 252-CMB-Symposium on Chemo Mechanical Characterization of Bituminous Materials.

[10] Zhang D., Chen M., Wu S., Liu Q., Wan J. (2018). Preparation of expanded graphite/polyethylene glycol composite phase change material for thermoregulation of asphalt binder. Constr. Build. Mater..

[11] Amin M., Putra N., Kosasih E.A., Prawiro E., Luanto R.A., Mahlia T. (2017). Thermal properties of beeswax/graphene phase change material as energy storage for building applications. Appl. Therm. Eng..

[12] Prabhu B., Gurusamy P., Arunkumar T. (2024). Solar photovoltaic cooling using Paraffin phase change material: Comprehensive assessment. Renew. Sustain. Energy Rev..

[13] Nazir H., Batool M., Osorio F.J.B., Isaza-Ruiz M., Xu X., Vignarooban K., Phelan P., Inamuddin, Kannan A.M. (2019). Recent developments in phase change materials for energy storage applications: A review. Int. J. Heat Mass Transf..

[14] Vasu A., Hagos F.Y., Mamat R., Kaur J., Noor M. (2019). The effect of thermal cyclic variation on the thermophysical property degradation of paraffin as a phase changing energy storage material. Appl. Therm. Eng..

[15] Du Y., Liu P., Wang J., Wang H., Hu S., Tian J., Li Y. (2019). Laboratory investigation of phase change effect of polyethylene glycolon on asphalt binder and mixture performance. Constr. Build. Mater..

[16] Chen M.Z., Hong J., Wu S.P., Lu W., Xu G.J. (2011). Optimization of phase change materials used in asphalt pavement rutting. Adv. Mater. Res..

[17] Sarı A., Sarı H., Önal A. (2004). Thermal properties and thermal reliability of eutectic mixtures of some fatty acids as latent heat storage materials. Energy Convers. Manag..

[18] Chen M., Zheng S., Wu S., Xu G. (2010). Melting intercalation method to prepare lauric acid/organophilic montmorillonite shape-stabilized phase change material. J. Wuhan Univ. Technol.-Mater. Sci. Ed..

[19] Chen J., Li J., Wang H., Huang W., Sun W., Xu T. (2019). Preparation and effectiveness of composite phase change material for performance improvement of Open Graded Friction Course. J. Clean. Prod..

[20] Wang X., Ma B., Yu M., Mao W., Si W. (2025). Testing and modeling of incomplete phase change heat storage and release of epoxy resin/microcapsule composite phase change materials for asphalt pavement. J. Energy Storage.

[21] Tian B., Yang W., Luo L., Wang J., Zhang K., Fan J., Wu J., Xing T. (2016). Synergistic enhancement of thermal conductivity for expanded graphite and carbon fiber in paraffin/EVA form-stable phase change materials. Sol. Energy.

[22] Amini N., Hayati P. (2020). Effects of CuO nanoparticles as phase change material on chemical, thermal and mechanical properties of asphalt binder and mixture. Constr. Build. Mater..

[23] Cheng C., Cheng G., Gong F., Fu Y., Qiao J. (2021). Performance evaluation of asphalt mixture using polyethylene glycol polyacrylamide graft copolymer as solid–solid phase change materials. Constr. Build. Mater..

[24] Guo M., Zhang S., Zhang R., Du X. (2024). Study on temperature regulation capability of asphalt mixture modified by dual phase change material used in ultra-thin overlay. Int. J. Pavement Eng..

[25] (2022). Plastics—Determination of Thermal Conductivity and Thermal Diffusivity—Part 2: Transient Plane Heat Source (Hot Disc) Method.

[26] Pyzalski M., Bialoskórski J., Walasek E. (1986). Reaction between carbon fibres and molten silicon: Heat determination using DTA. J. Therm. Anal..

